# OReO: optimizing read order for practical compression

**DOI:** 10.1093/bioadv/vbaf128

**Published:** 2025-06-03

**Authors:** Mathilde Girard, Léa Vandamme, Bastien Cazaux, Antoine Limasset

**Affiliations:** Univ. Lille, CNRS, Centrale Lille, UMR 9189 CRIStAL, F-59000 Lille, France; Univ. Lille, CNRS, Centrale Lille, UMR 9189 CRIStAL, F-59000 Lille, France; Univ. Lille, CNRS, Centrale Lille, UMR 9189 CRIStAL, F-59000 Lille, France; Univ. Lille, CNRS, Centrale Lille, UMR 9189 CRIStAL, F-59000 Lille, France

## Abstract

**Motivation:**

Recent advances in high-throughput and third-generation sequencing technologies have created significant challenges in storing and managing the rapidly growing volume of read datasets. Although more than 50 specialized compression tools have been developed, employing methods such as reference-based approaches, customized generic compressors, and read reordering, many users still rely on common generic compressors (e.g. gzip, zstd, xz) for convenience, portability, and reliability, despite their low compression ratios. Here, we introduce Optimizing Read Order (OReO), a simple read-reordering framework that achieves high compression performance without requiring specialized software for decompression. By grouping overlapping reads together before applying generic compressors, OReO exploits inherent redundancies in sequencing data and achieves compression ratios on par with state-of-the-art tools. Moreover, because it relies only on standard decompressors, OReO avoids the need for dedicated installations and maintenance, removing a key barrier to practical adoption.

**Results:**

We evaluated OReO on both Oxford Nanopore Technologies (ONT) and HiFi genomic and metagenomic datasets of varying sizes and complexities. Our results demonstrate that OReO provides substantial compression gains with comparable resource usage and outperforms dedicated methods in decompression speed. We propose that future compression strategies should focus on reordering as a means to let generic compression tools fully exploit data redundancy, offering an efficient, sustainable, and user-friendly solution to the growing challenges of sequencing data storage.

**Availability and implementation:**

The OReO code is open source and available at github.com/girunivlille/oreo.

## 1 Introduction

Over the past decade, high-throughput sequencing technologies have transformed sequence bioinformatics into one of the most data-intensive scientific fields. Modern sequencers can now generate tens of terabases of data per day, enabling unprecedented scientific discoveries while simultaneously creating formidable challenges in data management and storage. Public repositories such as the Sequence Read Archive and the European Read Archive reflect this explosive growth as they already house over 60 petabases of data, and these archives continue to expand at an accelerating pace. Despite the inherent redundancy in most sequencing data, their compression remains nontrivial for several reasons. First, the sheer volume of reads necessitates handling extremely large files. Second, shared sequences are often dispersed throughout a dataset, making it difficult for compressors to retain the massive context needed to exploit these redundancies. Third, sequencing errors introduce noise that hampers the detection of common subsequences and they themselves are relatively incompressible. Consequently, generic compressors frequently struggle to achieve better than a four-fold reduction, effectively storing each nucleotide in two bits. In response, researchers have developed a plethora of specialized methods to handle the unique properties of sequencing data, which can be broadly categorized as follows: Tuned generic compressors, Reference-based compressors, and Reordering-based compressors.

### 1.1 Tuned generic compressors

A number of specialized tools build upon established compression techniques but tailor them to the distinctive characteristics of sequencing data. For instance, DSRC (Deorowicz and Grabowski 2011), BIND (Bose *et al.* 2012), and DELIMINATE (Mohammed *et al.* 2012) employ a LZ77-style dictionary to capture recurring patterns within large datasets, while Fastqz (Bonfield and Mahoney 2013), LFQC (Nicolae *et al.* 2015), and LFastqC (Al Yami and Huang 2019) leverage the PAQ compressor family. Similarly, Fqzcomp (Bonfield and Mahoney 2013) and GTZ (Xing *et al.* 2017) take advantage of arithmetic coding, and Fqzcomp additionally includes a *k*-mer model to predict subsequent nucleotides, thereby improving compression efficiency. Other tools incorporate hybrid strategies tailored to specific components of the FASTQ format (headers, sequences, and quality scores), as seen in kungfq (Grassi *et al.* 2012), DSRC2 (Roguski and Deorowicz 2014), and FQC (Dutta *et al.* 2015). G-SQZ (Tembe *et al.* 2010) applies Huffman encoding by associating each base with its corresponding quality value, whereas NAF (Kryukov *et al.* 2019) takes advantage of modern compressors such as zstd. Some approaches, notably MFcompress (Pinho and Pratas 2014), rely on multiple competing finite-context models to efficiently capture both nucleotide and quality information. Finally, SolidZipper (Jeon *et al.* 2011) and SeqDB (Howison 2013) prioritize decompression speed by employing interleaved block compression, demonstrating that tuned compressors can combine compactness with practical performance characteristics.

### 1.2 Reference-based approaches

A second major class of tools exploits the fact that most reads can be aligned to a known reference genome, thereby encoding only positions and discrepancies. This strategy can be extremely space-efficient when the selected reference closely matches the read set. However, it also has inherent constraints: a suitable reference may be unavailable (e.g. for metagenomic datasets or previously unsequenced organisms), or it may be insufficiently similar if the organism exhibits high polymorphism. Moreover, compression methods that rely on an external reference can be cumbersome to decompress unless the reference is stored within the compressed file, which itself increases file size. Several tools rely on such alignment-based compression. For example, Gencompress (Daily *et al.* 2010), CRAM (Fritz *et al.* 2011), Fastqz (Bonfield and Mahoney 2013), and FQZip (Zhang *et al.* 2015a) map each read to a known reference, storing only the variations. Other methods, such as SlimGene (Kozanitis *et al.* 2010), SamZIP (Sakib *et al.* 2011), NGC (Popitsch and von Haeseler 2013), Gobi (Campagne *et al.* 2013), and LW-FQZip (Zhang *et al.* 2015b), compress the SAM files resulting from these alignments. More recent tools propose advanced strategies, HUGO (Kingsford and Patro 2015) handles unmapped reads by searching across multiple reference genomes, while Deez (Hach *et al.* 2014) locally assembles unmapped reads before compressing them. BARCODE (Rozov *et al.* 2014) avoids the explicit mapping step by inserting reads into a Bloom filter cascade and using the reference sequence to query these filters. Meanwhile, kpath (Kingsford and Patro 2015) uses the reference to build a de Bruijn graph and encode reads according to the paths they traverse in the graph. Interestingly, some tools implement “de novo” versions of reference-based compression by constructing their own internal references. Quip (Jones *et al.* 2012), for instance, builds a reference sequence directly from the input data and maps reads to it, whereas Leon (Benoit *et al.* 2015) generates a probabilistic de Bruijn graph as a surrogate reference and encodes reads accordingly. These adaptations expand the applicability of reference-based methods by mitigating some of the practical drawbacks associated with external reference genomes.

### 1.3 Reordering approaches

A third family of compression strategies leverages the fact that the order of reads in most sequencing datasets is unimportant to downstream analyses. By rearranging reads so that overlapping or highly similar sequences are placed in close proximity, these methods enable more efficient “local” pattern detection without requiring compressors to maintain a large global context. Although some downstream analysis assume uniform read distributions that could be hindered by such reordering, this can be fixed after decompression by shuffling the reads. Many tools implement reordering-based compression. Coil (White and Hendy 2008) and Recoil (Yanovsky 2011) cluster reads with significant suffix–prefix overlaps, while BEETLE (Cox *et al.* 2012) optimizes the read order for Burrows-Wheeler Transform (BWT)-based compression. Scalce (Hach *et al.* 2012) applies Locally Consistent Parsing (LCP) to improve the performance of generic compressors like gzip. ORCOM (Grabowski *et al.* 2015) and Mince (Patro and Kingsford 2015) organize reads according to minimizers, a concept extended by BdBG (Wang *et al.* 2018) via path-encoding strategies that echo those of Quark and Leon. Meanwhile, Harc (Chandak *et al.* 2018) and Minicom (Liu *et al.* 2019) reorder reads according to contigs assembled from the input, and Assembltrie (Ginart *et al.* 2018) constructs a “read forest” reference to guide reordering. Fastore (Roguski *et al.* 2018) combines initial minimizer-based binning with a final ordering pass reminiscent of Harc, and FQSqueezer (Deorowicz 2020) relies on a *k*-mer dictionary to implement a dynamic Markov coder for partial-matching predictions. PgRc (Kowalski and Grabowski 2020) approximates a shortest common superstring to serve as a central reference [with a GPU-based variant provided by CURC (Xie *et al.* 2022)], while Mstcom (Liu and Li 2021) attempts to construct a minimum spanning tree to represent how reads can be differentially encoded. Finally, Quark (Sarkar and Patro 2017) proposes a semi-reference-based strategy, in which a reference is required only during compression but not decompression. Collectively, these approaches illustrate how optimized read reordering can dramatically improve compression efficiency by amplifying local sequence similarity.

### 1.4 Related work

Although most compression efforts focus on a single read set, some methods exploit shared redundancy by co-compressing multiple *related* datasets. For example, DARRRC (Holley *et al.* 2018) encodes a de Bruijn graph with Bloom filters to jointly compress many datasets, while PMFFRC (Sun *et al.* 2023) uses clustering of reads from different collections to optimize co-compression. MRC (Tang and Li 2021) applies unsupervised learning for similar purposes, and Mizar (Pratas and Pinho 2024) groups metagenomic datasets prior to compression by leveraging taxonomic assignment. Outside the main scope of this work, some tools adopt a *lossy* paradigm, where certain bases are altered to improve compressibility. Because stochastic sequencing errors are inherently costly to represent, fixing them can reduce file size significantly. BFQzip (Guerrini *et al.* 2022), for instance, modifies bases to improve compression efficiency, demonstrating that lower error rates typically enable better compression, albeit at the potential cost of introducing analysis bias. A final, extensive line of research focuses on *quality scores*, which can be difficult to compress yet are useful for some downstream tasks as genotyping. Several studies demonstrate that discretizing (Wan *et al.* 2012) quality scores can yield substantial compression gains with only a minimal effect on downstream analysis. However, those subjects lie beyond the scope of our present discussion.

### 1.5 Long reads

At the onset of third-generation sequencing technologies, long-read datasets were relatively rare and offered lower coverage, in large part due to higher costs. In addition, error rates exceeding 10% posed extra challenges for compression by introducing substantial noise, making these datasets less amenable to efficient encoding. However, with recent cost reductions and error rates now approaching those of second-generation sequencing platforms (Bankevich *et al.* 2022, Sanderson *et al.* 2023), the compression of long-read data has become increasingly relevant, and several new tools have emerged to address this need. ENANO (Dufort y Álvarez *et al.* 2020) is a tuned generic compressor relying on arithmetic encoding. RENANO (Dufort y Álvarez *et al.* 2021) is more closely aligned with reference-based strategies but operates both with and without an external reference by performing de novo assembly of the input reads to compensate for its absence. NANOSPRING (Meng *et al.* 2023) follows a similar approach, using MinHash sketches to assemble the reads and then encoding them according to the constructed reference. Because the reads are reordered based on their positions in the assembled contigs, NANOSPRING is also related to reordering-based methods. Other reordering-based compressors include FastqCLS (Lee and Song 2022), which reorders reads based on nucleotide distribution rather than overlap, and Colord (Kokot *et al.* 2022), which computes *k*-mer similarity among reads to encode them relative to one another. Interestingly, when these compressors internally reorder the reads, they typically record the original order so that the file can be reconstructed exactly upon decompression. This approach diverges somewhat from standard second-generation sequencing (NGS) compression, largely because the overhead for storing positions is relatively small: preserving the order of *N* reads requires about N log(N) bits. For example, in a large mammalian dataset with 10 million reads of length 10 kb (totaling 100 gigabases), storing the positions with 32-bit integers amounts to approximately 40 MB, which can be reduced to around 30 MB using entropy-based compression. This overhead is minimal when compared to the total data volume. Furthermore, as read lengths increase, fewer total reads are necessary to cover the same number of bases, which further diminishes the storage costs associated with preserving their original order.

### 1.6 Going beyond dedicated compressors

A high-level comparison of the three main compression paradigms reveals that reference-based methods generally achieve impressive compression ratios but depend on an external reference, tuned generic compressors can operate *de novo* albeit at lower efficiency, and reordering approaches appear to offer the best of both worlds by providing strong compression without relying on a reference. In this article, we address the critical issue of dedicated compressors: although more than 50 specialized compression tools have been developed, their actual impact on day-to-day data storage and handling remains minimal. Instead, end users typically rely on generic compressors like gzip, zstd, or xz. Several factors contribute to this preference:

- **Ease of use:** Generic tools require no additional installations or configuration steps.
- **Portability:** These tools are readily available on all major operating systems and High-Performance Computing (HPC) environments.
- **Compatibility:** Generic compressed files are easily handled by other tools and workflows due to widely available C++ and Python libraries.
- **Maintainability:** Many academic compressors lack ongoing support and updates, whereas generic tools are maintained and validated by large communities.

Given these barriers, we propose a shift in perspective: rather than developing yet another custom compressor, *we improve the datasets compressibility* for existing generic tools. Specifically, we rely on well-established reordering techniques to rearrange reads such that those sharing similarities are placed in close proximity, thereby producing semantically equivalent files that are much more amenable to generic compression. Focusing on *pre-compression*, i.e. preprocessing the data before applying a standard compressor, offers several advantages. First, using widely accessible tools eliminates concerns about software availability, reliability, and long-term maintenance. Second, as we will show, this approach can deliver compression ratios comparable to state-of-the-art dedicated methods, representing an enormous gain over naive usage of generic compressors. Third, while generic compressors often provide fast decompression, reordering pre-processing can further enhance decompression throughput in some scenarios and vastly outperform dedicated compressors. In the following, we introduce a proof-of-concept tool, OReO, which reorders reads based on a draft assembly graph. Our design emphasizes simplicity and modularity, leveraging a Python implementation that integrates well-known, tunable third-party utilities. This highlights that reordering long reads need not be inherently complex and that straightforward methods can yield excellent results. We then benchmark OReO against specialized long-read compressors (NanoSpring and CoLoRd) and generic tools (gzip, zstd, bzip2, and xz), both with and without reordering, on genomic and metagenomic data with both Oxford Nanopore Technologies (ONT) and HiFi datasets spanning various genome sizes and complexities. We did not include compressors based on short-read reordering, as they assume all reads have the same length, an assumption that does not hold in our long-read datasets. In addition, we excluded FastqCLS, as its compression performance is effective only on FASTQ format datasets and performs poorly on FASTA files compared to NanoSpring and Colord after being tested on our datasets. Our experiments show that generic compressors, when coupled with reordering, can achieve near state-of-the-art compression rates alongside fast decompression. Finally, we examine potential ways to improve resource usage and file-size efficiency, drawing on earlier research into compression-resource trade-offs.

## 2 Method

### 2.1 High-level overview

**Figure 1.**
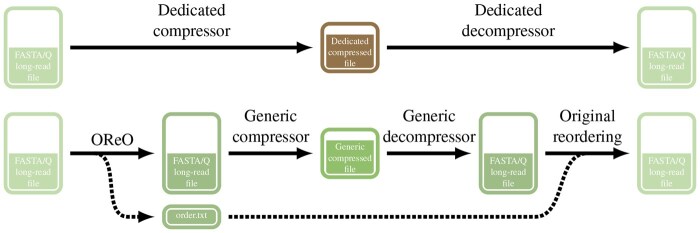
OReO’s use case contrasted with specialized compression tools.

We introduce OReO, a tool that reorders FASTA long-read files so that reads with overlapping sequences appear in close proximity (see Fig. 1). By approximating their natural genomic arrangement, OReO ensures that overlapping reads, which share substantial subsequences, are placed nearby. In practice, this strategy enhances generic compression tools, as they can more efficiently exploit local redundancies. Because reordering alone does not modify any sequence data, the resulting compression can be considered lossless. Moreover, OReO does not depend on a specialized compression format, nor does it require an external reference genome. These features make it straightforward to integrate OReO into existing workflows and to pair it with standard, well-maintained compressors already familiar to most users.

**Figure 2.**
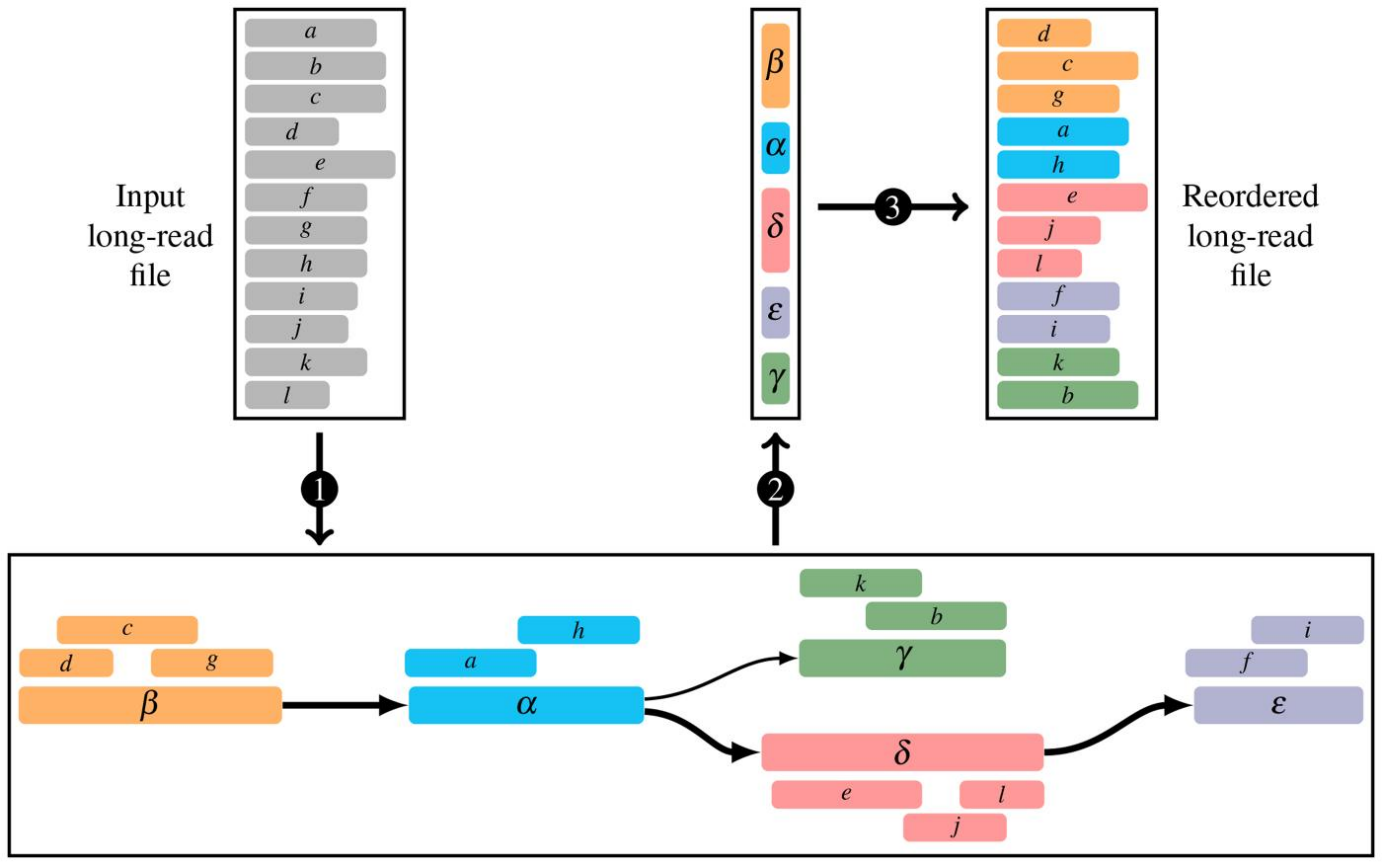
Overview of OReO’s reordering process for long-read data. The workflow involves (1) generating a contig graph, (2) traversing this graph to determine the order of contigs, and (3) reordering reads according to their positions on each contig.

OReO follows three main steps to sort a file (see Fig. 2):

1. *Genome assembly*—generate a contig graph from input reads,
2. *Contigs ordering*—use a graph traversal to order contigs,
3. *Read placement*—order reads using the order of contigs obtained and the positions of reads on each contig.

The output file contains the same reads as the input and can then be compressed with any generic compressor.

### 2.2 Genome assembly

Constructing a *de novo* assembly and mapping the reads to it is arguably the most straightforward method to order reads by their mutual relationships, and this approach has been employed by multiple tools for both second- and third-generation sequencing datasets. Numerous long-read assemblers are available in the literature, which aim to produce high-quality, error-free genomes but often demand substantial runtime and memory resources. Here, our goal is merely to obtain an approximate, “noisy” assembly that allows us to map and order the reads effectively, rather than to produce a biologically accurate consensus sequence. Consequently, high precision is not essential, and we prioritize speed over accuracy. We therefore adopt miniasm (Li 2016), a simple string graph assembler that avoids expending computational resources on complex repeat resolution or error correction. In OReO, the required all-versus-all alignments are carried out using minimap2 (Li 2016). Because OLC-based assemblies can become expensive at very high coverage, the user may optionally specify a subsampled set of reads (ideally 30–60×) to reduce both runtime and resource usage during assembly. Including at least 30× coverage of the longest reads is recommended to ensure that the resulting assembly remains sufficiently contiguous. Miniasm ultimately produces contigs in FASTA format, which serve as the basis for aligning and ordering the full set of reads. Practically, parameters for both minimap2 and miniasm can be tuned to account for specific read properties (e.g. length or error rate), optimizing memory usage and runtime without compromising assembly contiguity. Nonetheless, we report OReO’s performance under its default settings, reported in the appendix, to illustrate its baseline capabilities.

### 2.3 Contig ordering

Ideally, the assembly process yields a small number of long, continuous contigs, thereby gathering reads that overlap one another in close proximity. However, unresolved repeats often cause assemblers to split the genome into multiple contigs. Typically, these contigs are output as an oriented graph in GFA format, where edges represent overlaps between contigs. Since reads sharing common subsequences end up in the same contig, and their divergent regions connect to other contigs, bringing linked contigs together also clusters overlapping reads. From contig connections, OReO reconstructs the contig graph and then employs a depth-first search (DFS) to traverse and order the contigs. DFS follows each path to its end before backtracking and exploring any remaining branches. This ensures that continuity between contigs is maintained wherever possible, while still including all contigs in the final ordering. The outcome is a contig order that reflects possible paths within the assembly graph, thereby grouping together reads with strong sequence similarity. In turn, these localized similarities improve the performance of subsequent compression steps, as reads that share overlapping regions are located closer to one another in the reordered file.

### 2.4 Read placement and ordering

To place reads in the final output file, they are first mapped onto the assembled contigs with minimap2. The resulting PAF file contains, among other details, the read and contig identifiers, the mapping start position on the contig, and the relative strand. OReO then sorts this PAF file by contig, and within each contig, by mapping start position. To generate the final ordered read set, OReO iterates through the contigs in the order determined by the assembly graph traversal. For each contig, it retrieves the associated reads from the sorted PAF file and writes them in ascending order of their alignment positions. Each read appears only once, the first time it is encountered. Unmapped reads that are missing from the PAF file are simply appended at the end of the output. The end result is a FASTA file that contains all input reads, but in an arrangement that places overlapping or similar reads close together.

### 2.5 Read orientation

By default, OReO writes all reads in the same orientation relative to their contig alignments. This choice is motivated by the fact that two reads, which originate from the same genomic region but are oriented differently, will compress poorly. Converting one read to the other’s orientation leads to more extensive redundancy, thereby enhancing compressibility. Moreover, a read and its reverse complement encode essentially the same information, since each strand represents the same DNA fragment. However, in certain analyses, such as the detection of strand-specific sequencing errors or structural variants, orientation can be biologically meaningful. To accommodate such use cases, OReO offers an option to preserve the original strand orientation. In this mode, each strand is handled as a different sequence: the mapping file is firstly separated by alignment strand orientation, then by contig and mapping position. As a result, reads that map to the reverse strand are neither reversed nor complemented; they remain in their original sequencing format. Because these orientation changes only affect how reads are represented, they are fully reversible if one maintains a small auxiliary file to track the original positions and orientations. For instance, an input with 10 million 10 kb reads would require storing a single integer (32 bits) per read to track read position (roughly 32 MB) and a single bit per read for orientation (roughly 1 MB). In practice, this overhead is minimal compared to the typical size of modern sequencing datasets.

## 3 Results

### 3.1 Experimental setup

All experiments were conducted on a single cluster node equipped with an Intel(R) Xeon(R) Gold 6130 CPU @ 2.10 GHz, 128GB of RAM, and running Ubuntu 22.04.

### 3.2 Dataset descriptions

*Escherichia coli*

HiFi: SRR11434954, 1.8M reads, 23.1 Gb (5000×). ONT: SRR28370668, 6.8M reads, 9.1 Gb (2000×).

*Arabidopsis thaliana*

HiFi: SRR14728885, 1.5M reads, 22.9 Gb (170×). ONT: SRR14739179, 3M reads, 56.8 Gb (420×).

Human

HiFi: SRR29483063–5, 11.4M reads, 194.1 Gb (60×). ONT: SRR23365080, 7.5M reads, 352 Gb (113×).

Zymo (Microbial Community)

HiFi: SRR13128014 (21 strains, 17 species), 2M reads, 18 Gb.

To better reflect commonly encountered coverage depths, we used the longest 100× reads (both HiFi and ONT) for *E. coli* and *A. thaliana*. For performance reasons, the human datasets were capped at 50×. Because the Zymo dataset features uneven coverage, the same subsampling strategy was inapplicable; thus, its results are also presented only in the Appendix. All results presented were processed on files in FASTA format.

### 3.3 Compressed size

**Figure 3.**
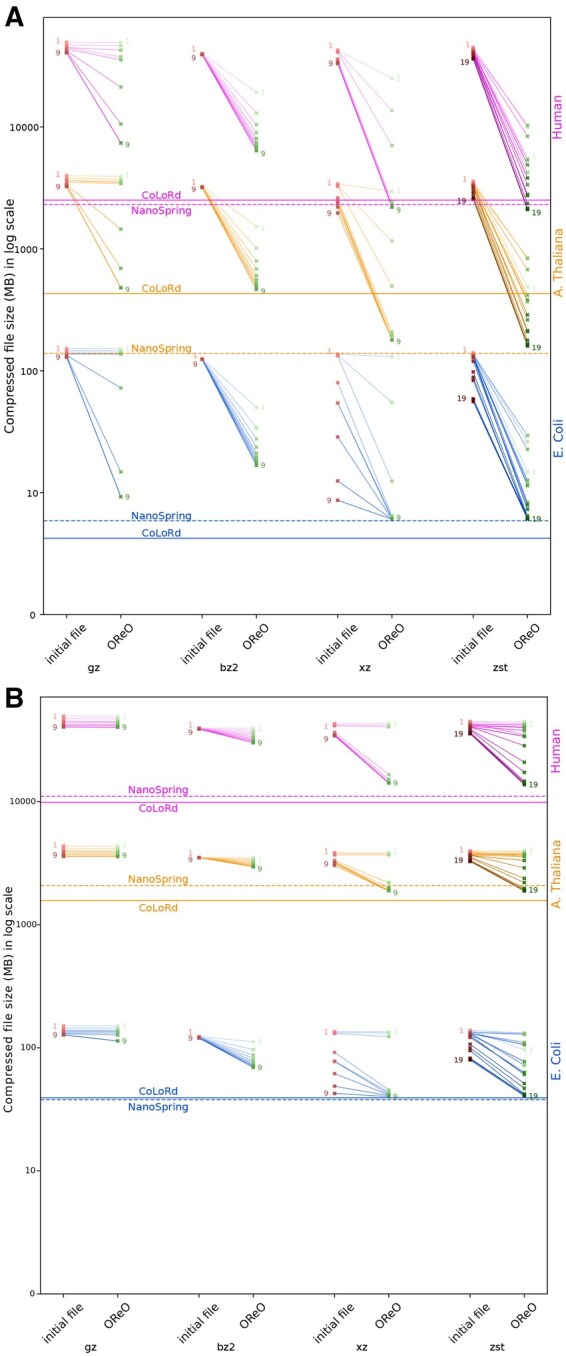
Comparison of file compression using various compressors and compression levels, both with and without OReO ordering, alongside CoLoRd and NanoSpring on (A) HiFi and (B) ONT datasets.

As an initial experiment presented in Fig. 3, we applied commonly used generic compressors (gzip, bzip2, xz, and zstd) to subsampled datasets from *E. coli*, *A. thaliana*, and humans, both *before* and *after* OReO reordering. Each compressor was tested across all available compression levels. We also evaluated the state-of-the-art compressors NanoSpring and CoLoRd; however, because these tools employ their own internal reordering strategies, OReO ordering does not affect them, so we report their performance only on the original, unreordered datasets. Surprisingly, although NanoSpring is intended to be lossless, its output file did not include the original read headers. Consequently, the comparison may not be entirely fair. Several observations can be made:

- *gzip* is widely used but often provides the lowest compression. With HiFi data, higher compression levels benefit from OReO ordering, though the effect is modest; for noisier ONT data, the improvement is barely noticeable. This suggests that gzip’s relatively small compression window rarely spans enough reads to exploit redundancy effectively.
- *bzip2* sees a consistent, albeit slight, improvement from OReO across all levels. Its overall compression ratio, however, remains less competitive than that of other compressors.
- *xz* is the only tool that achieves notable compression on the original datasets, especially for smaller genomes, indicating its ability to handle large contexts at higher compression levels. OReO substantially boosts its performance, even surpassing dedicated compressors at some high compression settings.
- *zstd* is less effective than xz on regular files, but delivers similar results on OReO-optimized files and can match or exceed dedicated compressors in some cases.
- *Noise level* As expected, the noisier ONT data are more challenging than their HiFi matches for all compressors.

Taken together, these results demonstrate that reordering can significantly enhance the performance of generic compression tools, occasionally matching or even outperforming specialized methods.

### 3.4 Decompression efficiency

**Figure 4.**
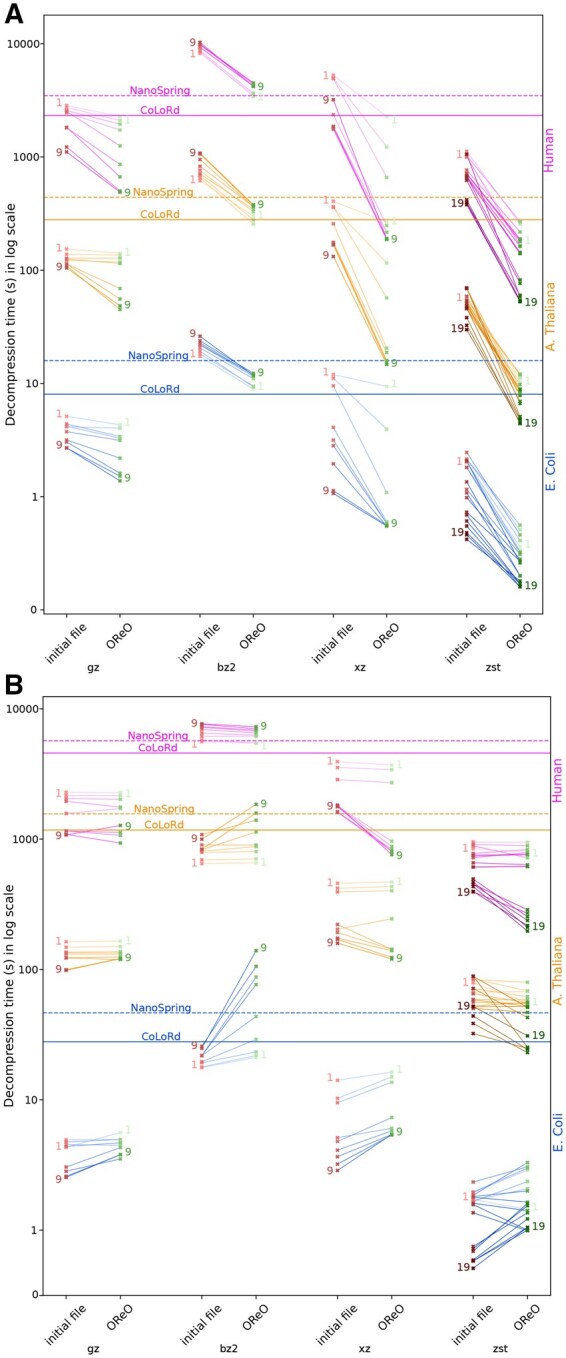
Decompression time benchmarks for 100× *E. coli*, *A. thaliana*, and *Human* datasets on (A) HiFi and (B) ONT.

We next evaluated the *decompression* performances in Fig. 4, which is often considered of higher practical importance than compression speed because files tend to be compressed only once but may be decompressed multiple times. In most cases, OReO-processed files decompress faster, sometimes by several-fold, particularly with xz and zstd. Moreover, generic decompressors operate considerably faster than dedicated decompressors, occasionally by an order of magnitude. Notable exceptions are bzip2, which can be slower than specialized tools and xz at lower compression levels. Overall, these observations highlight another practical benefit of relying on generic (de)compressors in conjunction with reordering: not only do they achieve competitive compression ratios, but they also offer consistently higher decompression speeds in everyday usage. RAM usage during decompression, reported in the appendix, shows that all generic compression tools exhibit negligible memory usage (<100MB), Colord displays low memory usage at decompression (<1GB) while NanoSpring can display high memory usage.

### 3.5 Compression efficiency

**Figure 5.**
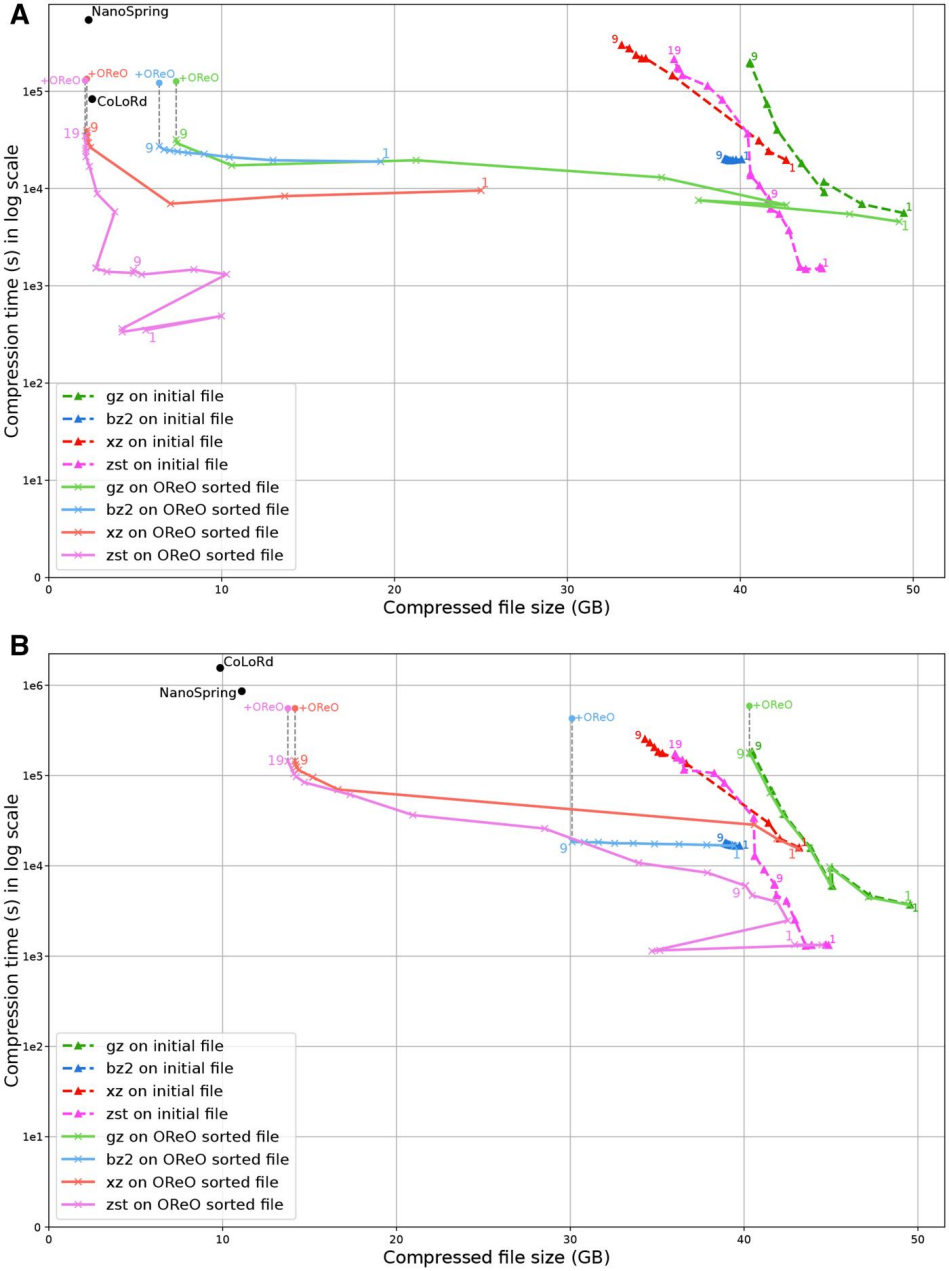
Compression time versus compressed file size for the human (A) HiFi dataset and (B) ONT dataset for initials and sorted files, with all compression levels. Preprocessing time is included by a dot for each sorted file compressed with the highest compression level.

In a third experiment, we evaluated the computational cost of various compression strategies and compared it with the resulting compression ratio. We present these results for the humans datasets in Fig. 5, and the other datasets can be found in the appendix. Across most datasets, reordering with OReO did not increase compression time; in some instances, it slightly reduced it, and in others, it led to a significant compression speed-up. Overall, all tools show rapid compression at lower settings, while higher gzip and zstd levels incur greater costs, and xz becomes especially expensive at its maximum setting. Despite the computational overhead of the required de novo assembly, combining OReO with a generic compressor generally results in faster runtimes than specialized tools like CoLoRd and NanoSpring. The only exception observed in our tests was the Human HiFi dataset. Detailed timing results are provided in the Appendix. RAM usage during compression, reported in the appendix, shows that all generic compressors display low memory usage (<1GB), while dedicated compressors and OReO can display high memory usage.

### 3.6 Impact of read orientation on compression

**Figure 6.**
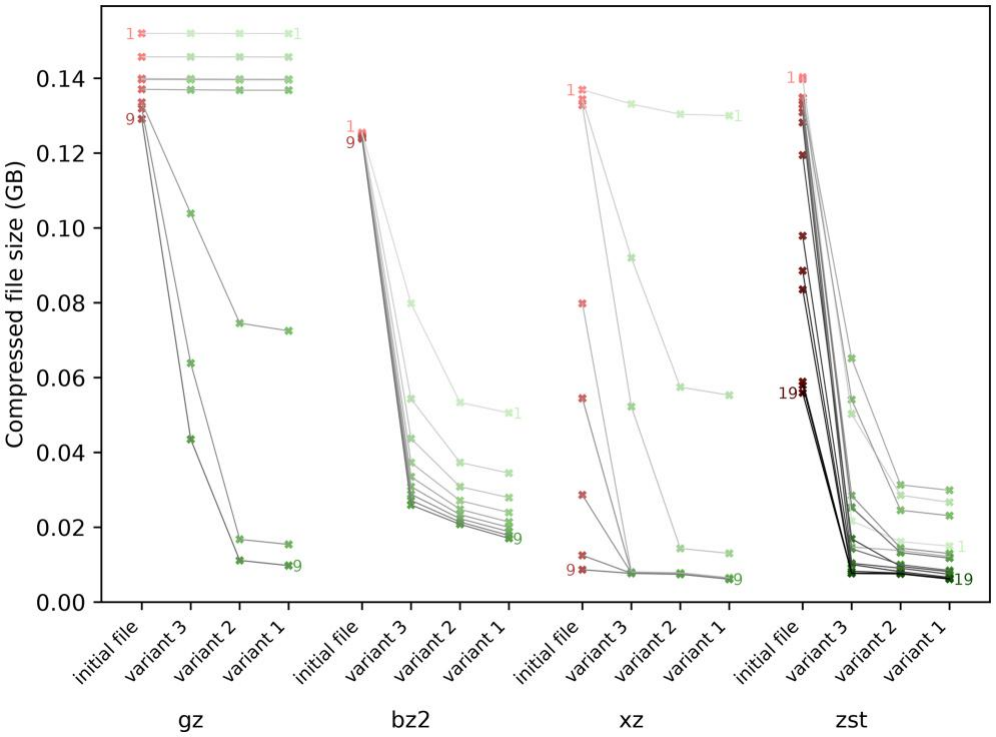
Comparison of three strategies for managing reverse-complement reads and their impact on compression efficiency. **Variant 1 (OReO default)** reorders reads and, when beneficial, reverse-complements them to maximize compression. **Variant 2** preserves the original read orientation, grouping same-strand reads together (OReO’s behavior when strand information must be retained). **Variant 3** reorders reads without considering their strand, typically resulting in lower compression efficiency.

Although reordering reads greatly improves compression for generic tools, strand orientation also plays an important role. To illustrate this, we compared three variants of OReO in Fig. 6:

1. **Optimizing both ordering and strand**, i.e. reordering reads and unifying their orientation (best compression).
2. **Optimizing only ordering while preserving strand**, i.e. reads remain in their original orientation but are reordered along other reads from the same strand (best choice when strand is biologically relevant).
3. **Optimizing only ordering without accounting for strand divergence**, i.e. ignoring strandness (worst compression).

Our results show that combining both strand unification and read ordering delivers the highest compression gains. When users wish to preserve the original strand, ordering each strand separately still yields considerable improvement, but neglecting strand orientation altogether can lead to degraded compression performance. Consequently, explicitly managing the reads strands, whether by unifying or separating them, must be factored in when using generic compressors, which (unlike most sequence analysis tools) do not inherently account for strand orientation.

### 3.7 Compressed index boosting

Beyond improving overall compression, OReO’s read reordering can also enhance indexing structures that rely on internal compression. One example is the K2R (*k*-mer to reads) index (Vandamme *et al.* 2025), which associates each *k*-mer with a compressed list of read identifiers containing that *k*-mer. With well-ordered reads, *k*-mers tend to be shared by neighboring reads, making their corresponding identifier lists more amenable to compression techniques such as delta encoding. To demonstrate this effect, we used K2R to index the previously described datasets with and without OReO preprocessing. As shown in Table 1, OReO’s reordering substantially improved both the index size and the construction runtime. Such properties could be leveraged in future index designs that aim to take full advantage of these overlapping and proximity-based features.

**Table 1.** K2R time usage and index size on various input file.

Dataset		Time (s)	Index (MB)
*E. coli* HiFi	Initial/OReO	114/**100**	60/**40**
*E. coli* ONT	Initial/OReO	112/**103**	60/**13**
*A. Thaliana* HiFi	Initial/OReO	3197/**2805**	1781/**367**
*A. Thaliana* ONT	Initial/OReO	3924/**3777**	2685/**2185**
*Human* HiFi	Initial/OReO	**21 951**/22 625	6959/**1731**
*Human* ONT	Initial/OReO	**36 718**/38 302	6997/**5095**

## 4 Future work

In this work, we presented OReO, a straightforward proof-of-concept tool that reorders reads according to a draft assembly before applying generic compressors. Despite its simplicity, this approach achieves compression ratios on par with state-of-the-art dedicated tools, yet requires comparable resources and offers faster and lighter decompression. We also demonstrated that such read ordering can improve the efficiency of certain index structures. We hope these results will encourage future developers to focus on read-reordering strategies rather than producing yet another specialized compressor, and to highlight for users that reordering offers substantial disk-space savings with almost no drawbacks. To provide assurances about data fidelity, we supply a built-in consistency check (similar to an MD5 hash) confirming that the reordered file preserves exactly the same sequences (or their canonical forms, if strand orientations are allowed to change). Additionally, an optional file storing the original read order and strand allows users to restore the input file if desired. The major limitation of our proposed workflow is its relatively high resource usage due to multiple read-mapping steps. In particular, the all-versus-all read comparison used for overlap-based assembly can be prohibitively costly and may not be necessary. Recent advances in genome assembly demonstrate that accurate drafts of long-read datasets can be generated without constructing overlap graphs, for example, by using de Bruijn-based frameworks with large *k*-mers or minimizer-based de Bruijn graphs (Ekim *et al.* 2021). One such approach, RMDBG (Ekim *et al.* 2021), was evaluated in our experiments but produced contigs too fragmented for our use case. A purpose-built greedy assembler, designed to prioritize computational efficiency over biological precision, could significantly reduce resource requirements. A second obstacle arises from the read-mapping step on the resulting draft assembly. This step recovers contig-read associations that the assembler itself already computes internally. Although tools like miniasm track some read information, they only retain data for the subset of reads used in assembly, discarding shorter reads. A custom assembler that explicitly records all read-contig links could eliminate the need for this secondary mapping step entirely. Beyond our current workflow, several alternative strategies could be explored. One possibility is a shortest-common-superstring approach, adapted to handle the intrinsic noise of raw reads. Methods that rely purely on sequence similarity, such as those using small *k*-mers, MinHash sketches, or minimizers, could also cluster reads more directly and potentially lower computational demands. However, despite its high resource requirements, OReO outperforms state-of-the-art tools in terms of speed on most datasets. Additionally, after just one run of OReO per dataset, the end user can make unlimited use of generic compressors and decompressors, benefiting from their low resource consumption. Another promising direction involves incorporating read-correction methods, which reduce noise in the input data and improve overall compression ratios. Nonetheless, some users are wary of error-correction tools because of the risk of introducing bias. A potential compromise is to store the differences between original and corrected reads, allowing the reconstruction of uncorrected reads. Even if these differences are difficult to compress, the rest of the data could benefit from significantly enhanced compressibility. The viability of this scheme, and whether its complexity is justified, remains an open question. Another way of improving compression ratios is to sort the unmapped reads. As shown in the appendix, unmapped reads represent only a small proportion of total reads in our datasets, so only a slight improvement is expected. However, we tested samtools sort with the option “-M” to sort unmapped reads on one dataset and included the results in the appendix. The gain is indeed slight and time cost is too high to adopt this method in practice in our current implementation. Also, the alignment-free methods mentioned earlier would eliminate the need to manage these reads. A possible further work is the management of FASTQ files. While OReO can sort FASTQ files, the compression benefit is less significant compared to FASTA files due to the incompressibility of quality scores. Improving FASTQ compression could involve extracting quality scores placed in the same order as their corresponding sequences, and compressing them separately, with or without loss. Finally, the co-compression of multiple similar datasets has yet to be explored for long reads. This represents an exciting challenge: developing methods to compress large collections of related files together while still enabling selective decompression of individual datasets without unpacking the entire collection.

## Data Availability

The identifiants of the SRA datasets that were used in the experiments are listed at the beginning of the Results section. The OReO code is open source and available at github.com/girunivlille/oreo.
